# Retrotransposon Insertion Polymorphisms (RIPs) in Pig Reproductive Candidate Genes

**DOI:** 10.3390/genes13081359

**Published:** 2022-07-28

**Authors:** Zhanyu Du, Enrico D’Alessandro, Emmanuel Asare, Yao Zheng, Mengli Wang, Cai Chen, Xiaoyan Wang, Chengyi Song

**Affiliations:** 1 College of Animal Science and Technology, Yangzhou University, Yangzhou 225009, China; dx120190120@yzu.edu.cn (Z.D.); asare.emmanuel175@yahoo.com (E.A.); mz120180996@yzu.edu.cn (Y.Z.); mx120200833@yzu.edu.cn (M.W.); 007302@yzu.edu.cn (C.C.); wxyan@yzu.edu.cn (X.W.); 2 Department of Veterinary Sciences, University of Messina, Via Palatucci snc, 98168 Messina, Italy; edalessandro@unime.it

**Keywords:** retrotransposon, polymorphism, pig genome, reproductive traits

## Abstract

Retrotransposons account for more than one-third of the pig reference genome. On account of the genome variability in different breeds, structural variation (SV) caused by retrotranspos-on-generated deletion or insertion (indel) may have a function in the genome. Litter size is one of the most important reproductive traits and significantly impacts profitability in terms of pig production. We used the method of bioinformatics, genetics, and molecular biology to make an analysis among different pig genomes. Predicted 100 SVs were annotated as retrotransposon indel in 20 genes related to reproductive performance. The PCR detection based on these predicted SVs revealed 20 RIPs in 20 genes, that most RIPs (12) were generated by SINE indel, and eight RIPs were generated by the ERV indel. We selected 12 RIPs to make the second round PCR detection in 24 individuals among nine pig breeds. The PCR detection results revealed that the RIP-A1CF-4 insertion in the breed of Bama, Large White, and Meishan only had the homozygous genotype but low to moderately polymorphisms were present in other breeds. We found that RIP-CWH43-9, RIP-IDO2-9, RIP-PRLR-6, RIP-VMP1-12, and RIP-OPN-1 had a rich polymorphism in the breed of Large White pigs. The statistical analysis revealed that RIP-CWH43-9 had a SINE insertion profitable to the reproductive traits of TNB and NBA but was significantly affected (*p* < 0.01) and (*p* < 0.05) in the reproductive traits of litter birthweight (LW) in Large White. On the other hand, the SINE insertion in IDO2-9 may be a disadvantage to the reproductive traits of LW, which was significantly affected (*p* < 0.05) in Large White. These two RIPs are significant in pig genome research and could be useful molecular markers in the breeding system.

## 1. Introduction

Elements containing reverse transcriptase genes are generally referred to as retrotransposons because they can move from one place to another in a genome by the reverse transcription of an RNA transposition intermediate. They are not the only transposable elements present in the genomes of eukaryotes. These are usually less abundant than retrotransposons and they are only represented by defective copies in humans and mice [1]. Retrotransposons occupy one-third to half of an organism’s genomes, which are dominated by long interspersed nuclear elements (LINEs) and short interspersed nuclear elements (SINEs), followed by long terminal repeat (LTR) retrotransposons [2]. In plants, LTR retrotransposons tend to be more abundant than non-LTR, thus about 40–70% of the total DNA in many crop plants has been identified to be LTR retrotransposons [3,4]. In pigs, Chen et al. [5] reported that retrotransposons accounted for 37.13% (929.09 MB) of their genome, whiles LINEs, LTRs, and SINEs accounted for 18.52%, 7.56%, and 11.05%, respectively.

Most of the retrotransposons are nested, mixed, inverted, or truncated in chromosomal sequences. Fragments of LTR with retrotransposon internal parts are located near other retrotransposons, which allows for the use of LTR sequences for PCR amplification. Genome sites with a high density of retrotransposons can be used to detect their chance association with other retrotransposons [6]. Different ways of using transposable elements as molecular markers have been designed. Their qualities such as abundance, general dispersion, and activity allow for the perfect conditions to develop molecular markers. Using retrotransposon sequences as molecular markers, many methods have been developed as primers in the polymerase chain reaction [7]. The inter-repeat amplification polymorphism techniques such as inter-retrotransposon amplified polymorphism (IRAP), retrotransposon microsatellite amplification polymorphisms (REMAP), or inter-MITE amplification have used abundant dispersed repeats such as the LTRs of retrotransposons and SINE-like sequences (inter-SINE amplified polymorphism—ISAP) [8].

Retrotransposons, as a good molecular marker insertion in the species’ genome, of different polymorphic genotypes in different individuals may have a specific function to their phenotypic characteristics. In plants, Xu and Ramakrishna [9] identified the phylogenetic distribution of LTR retrotransposon, LINE, and SINE insertions in six genes in 95 cultivated and wild rice genotypes. In addition, the insertion of a retrotransposon could cause a phenotypic change in the skin color of the grapes to white. This is partly due to a retrotransposon in the promoter of *VvMYBA1*, one of the two regulatory genes controlling anthocyanin biosynthesis [10]. Proceeding with sequencing the candidate gene *OCA2* in the uncovered genomic interval, Suzanne et al. [11] identified that the insertion of an LTR-retrotransposon in its 11th intron resulted in a considerable truncation of the phloem (P) protein and is likely constituted to the causal mutation of a melanism in corn snakes. Similarly, in animals, Song et al. [12] found an L1 retrotransposon insertion-induced deafness mouse model to study the development and function of the cochlear stria vascularis. Yamamoto et al. [13] found that SVA retrotransposon insertion in the exon of *MMR* genes resulted in aberrant RNA splicing and causes Lynch syndrome.

The major goal of animal production is to improve reproductive efficiency. Reproductive physiology traits are of great economic importance since an increase in the litter size can significantly affect profitability [14]. Although reproductive traits are complex, desirable reproductive phenotypes such as the litter size are true polygenetic traits affected by interactions between multiple genes and the environment [15]. The use of a genome-wide association study (GWAS) in a Duroc pig herd to examine the reproductive traits such as litter size at birth (LSB), litter weight at birth (LW), litter size at weaning (LSW), and litter weight at weaning (LWW) revealed that several candidate single-nucleotide polymorphisms (SNPs) and genes were found to be potentially associated with the traits of interest [16]. Recently, Chen et al. [17] conducted genome scans of epistatic interactions underlying the litter size at birth (TNB) and the number of piglets born alive (NBA) traits in indigenous Chinese pigs (Jinhua and Shengxian Spotted pigs) with high throughput genomic data, and based on SNPs with high interaction values and connectivity scores, identified eight promising candidate genes potentially associated with the litter traits in pigs.

Genetic improvement programs have led to moderate gains in litter size traits because of their low heritability, strong heterosis, sex and adult limited measurements [18]. However, we could apply retrotransposon insertion polymorphic (RIPs) to make detection in different pig breeds’ genome, which will lead to a complementary tool in implementing gene marker assisted selection. In addition, according to the literature on reproductive efficiency genes in pigs, 20 associated genes were selected to identify the structural variations (SVs), which are derived from retrotransposons in Repeat Masker annotated pig genomes. The predicted results were then further confirmed by standard PCR and identified with genotypic distributions. It also contributes to a better understanding of the differences between these polymeric molecular markers in pig breeds and provides a theoretical basis for subsequent studies.

## 2. Materials and Methods

### 2.1. Ethical Statement

Animals were handled humanely for biological sample collections. The experimental procedures involved in this study were in accordance with the Animal Experiment Ethics Committee of Yangzhou University (No. NSFC2020-dkxy-02, 27 March 2020).

### 2.2. Sequence Acquisition for Reproductive Efficiency Genes

The sequence information of *BF*, *BRCA1*, *EPOR*, *FSHB*, *FUT1*, *GNRHR*, *IDO2*, *LEP*, *OPN (SPP1)*, *PRLR*, *Rab2A*, *RBP4*, *CASP6*, *CWH43*, *P2RX3*, *ZNF518A*, *UCHL1*, *VMP1*, *RAB1FIP4*, and *A1CF* are shown in Table S1.

The extension of 1000 bp on both sides of each sequence was used to Blast the WGS to define the same genomic positions for the 16 assembled nonreference genomes. These genomes represent the types of pigs used: Tibetan pig, Wuzhishan, Bama, Duroc, Hampshire, Berkshire, Pietrain, Landrace, Yorkshire, Yorkshire & Landrace & Duroc, Bamei, Jinhua, Rongchang, Meishan, Duroc-ref, and Duroc-Ninghe (detailed information list in NCBI was acquired at https://www.ncbi.nlm.nih.gov/genome/browse/#!/eukaryotes/84, accessed on 4 July 2022).

### 2.3. Structural Variation Prediction, Retrotransposon Annotation, and Insertion Polymorphic Prediction

The SVs prediction results were obtained by sequences of multiple alignments with Clustalx software [19], viewed by Jalview, Bioedit, and Sublime 4, and annotated by Repeat Maker [20]. The size of SVs beyond 50 bp were recorded for further analysis. Filter criteria for annotation results required exhibiting a cutoff score of more than 1000 bp for the masking repeats.

### 2.4. Animals for RIPs Verification and Genotyping

Twelve domestic pig breeds (Duroc, Landrace, Large White, Meishan, Erhualian, SuJiang, SuShan, Bama, Tibetan, Wuzhishan, BBY, and Wild-breed) were selected for the initial round of breed detection using the standard PCR. Pigs were clustered, thus three individuals per cluster for RIP detection. The clusters included (i) Meishan, SuJiang, Erhualian, and Sushan from Jiangsu Province; (ii) Bama, Wuzhishan, and Tibetan from the Guangxi, Hainan, and Sichuan Provinces, respectively, and (iii) Landrace, Yorkshire, Duroc pigs, and BY from our lab were collected from Anhui Province. A further, 24 individuals per breeds (Duroc, Meishan, Erhualian, Fengjing, Large White, SuJiang Sushan, Bama, and Tibetan) were also selected and clustered for the second-round breed detection by standard PCR. The wild-pig breed was selected from the Anhui and Heilong Jiang Provinces, respectively.

### 2.5. Samples Collection and PCR Analysis

All experimental pigs were more than 6 months old. Samples were collected from the ear tissue using standard procedures. The total DNA was extracted using the Takara DNA Extraction Kit following the manufacturer’s instructions (TaKaRa, Dalian, China). The gene special primer pairs designed based on the reference pig genome from NCBI (Sscrofa11.1) and the Oligo 7.0 software. PCR amplifications were carried out in a total volume of 20 µL (at least 50 ng of DNA content), Taq Master Mix buffer (Vazyme, Nanjing, China). The PCR reaction environment and conditions at 95 °C for 5 min, followed by 30 cycles of 95 °C for 30 s, 58 °C for 20 s, and 72 °C for 30 s, and a final extension of 5 min at 72 °C. DL2000 as DNA marker (TaKaRa, Dalian, China).

### 2.6. PCA and Cluster Analysis of the SINE RIPs

Based on the SINE RIPs identified in this study, the R statistics package (version 3.6.3, Robert Gentleman and Ross Ihaka, Auckland, can be found at the website of https://www.r-project.org/contributors.html, accessed on 4 July 2022) was used to generate a presence/absence matrix and perform the PCA analysis. On the same dataset, heatmaps and cluster analysis were computed by the use of the R package heatmap tool (version 1.0.12, Raivo Kolde, Republic of Estonia, can be found at the website of https://www.rdocumentation.org/packages/pheatmap/versions/0.7.2, accessed on 4 July 2022) [21] using the “Euclidean” distance method for clustering.

### 2.7. Traits Determinant

We collected 252 female pig individuals of Large White ear samples, which definitely had reproductive characteristic related records in the same year. The multiparity pig individuals used to conduct the statistical analysis came from the same farm and had no relationship with each other. The samples’ phenotypic characteristics, traits, and determinant were highly standardized including the first total number born (TNB), first number born alive (NBA), and litter birthweight (LW) in the first parity.

### 2.8. Statistical Analysis

The experimental results were processed by the nonparametric test pairwise comparison of independent samples [22] using the statistical SPSS17.0 software package (SPSS, Inc., Chicago, IL, USA) and the data were expressed as the mean ± S.D. In this trial, each pig served as the experimental unit. Statistical differences were considered significant at *p* < 0.05. and *p* < 0.01 was considered to be extremely significant.

## 3. Results

### 3.1. Reproductive Related Genes’ SVs Revelation and Detection by Pool-PCR in Different Pig Breeds

The sequences of reproductive related genes and their flanking regions were reassembled based on the NCBI genome deposited sequences. The detailed information of the flanking sequences of the candidate genes are summarized in Table S1. We obtained 100 large SVs of reproductive related genes (note; size larger than 50 bp) as putative RIPs, which are summarized in Table 1 and Table S2.

**Figure 1:**
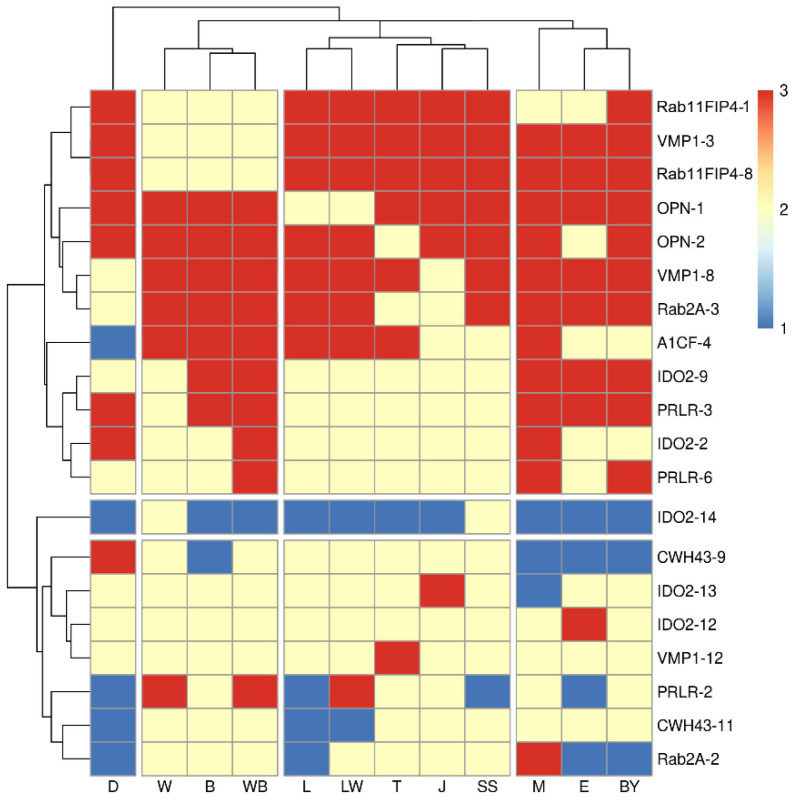
The heatmap results for the pool-PCR of D (Duroc), W (Wuzhishan), B (Bama), WB (Wild-breed), L (Landrace), LW (Large White), T (Tibetan), J (SuJiang), SS (SuShan), M (Meishan), E (Erhualian), and BY (BY). We defined blue block as homozygous insertion, yellow block as heterozygosis insertion, and red block as homozygous deletion in the genome of different pig breeds.

The 100 RIPs were detected by standard PCR from the wild pigs’ genomic DNA and eleven domesticated pig breeds. At the end, 20 RIPs were confirmed, and the results were shown in Figure 1 and Figure S1, and Table S3. However, the SVs were not encountered in the *UCHL1* gene, and among these RIP predictions, 5, 3, and 3 confirmed the RIPs of IDO2, *PRLR*, and *VMP1* respectively, and most results were detected by pool-PCR.

After transforming, the pool-PCR electrophoretic results were numerated and the data were processed to make a clustering analysis. The results were clustered into three gene-row parts, and the breeds column into four parts, thus (i) the breeds of Meishan and Erhualian; (ii) Wuzhishan and Bama; (iii) Landrace, Large White, Sujiang, and Sushan together; (iv) the breed of Duroc as standalone. We defined the red blocks as homozygous deletion enriched on the top, the blue blocks as homozygous insertion enriched at the bottom, and the other yellow blocks were heterozygosis insertion. We discovered that nearly 60% of these RIPs had insertion including the homozygous inserted type and heterozygosis inserted type, which was more relevant in our study. This accounts for the fact that the 60% RIPs used in the detection of small groups of individuals were actually used to check for the existence of the three genetic individuals in the special breed.

**Figure 2:**
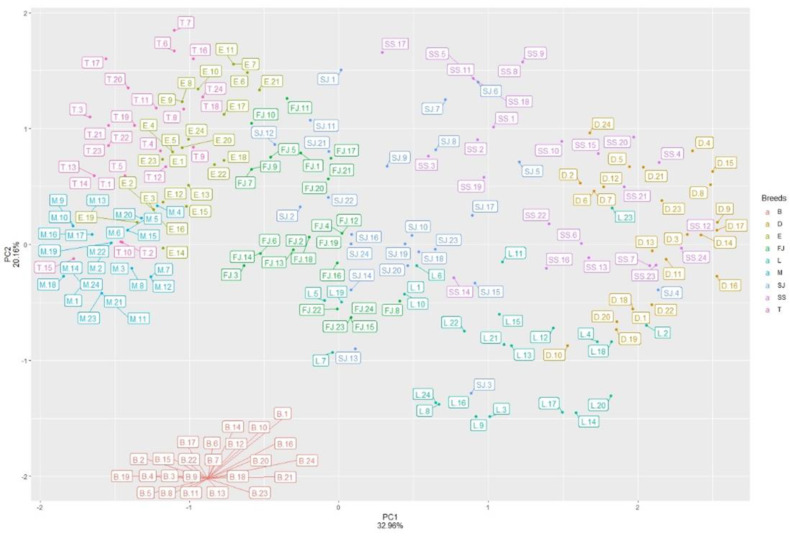
The principal component analysis (PCA) based on the results of the 24 individuals per breed. “B”, the Bama breed; “T”, the Tibetan breed; “D”, the Duroc breed; “L”, the Large White breed; “SJ”, the SuJiang breed; “SS”, the SuShan breed; “E”, the Erhualian breed; “M”, the Meishan breed; “FJ”, the Fengjing breed.

The 12 RIPs of the first-round pool-PCR detected results were chosen as the second-round PCR detected site, which showed undoubled polymorphism types in twelve pig breeds and detected in 24 individuals for nine breeds. The information of these 12 predicted RIPs are shown in Table 2. The electrophoresis results and the number format are illustrated in Figures S2–S5 and Table S4. Next, we conducted principal component analysis (PCA), which was clustered in Chinese indigenous pig breeds (Meishan, Erhualian, FengJing, and Tibetan) at the left of Figure 2, and commercial breeds (Duroc, and Large White) on the right. In addition, SuShan and SuJiang, as the hybridized breeds, were clustered at the center section. There was only one genotype in the breed of Bama per RIPs, which was clustered at the bottom.

### 3.2. Statistical Analysis for the RIPs with the Reproductive Characteristic of Large White Pig

After 20 RIPs were determined in 24 individuals of pig breeds including Bama, Tibetan, Duroc, Large White, SuJiang, SuShan, Erhualian, Meishan, and FengJing, we found that RIP-CWH43-11, RIP-OPN-2, RIP-PRLR-2, RIP-PRLR-3, and RIP-Rab11FIP4-1 had no polymorphism distribution within each breed. However, the RIP-A1CF-4 insert in the Bama, Large White, and Meishan breeds only had the homozygous genotype, but low to moderately polymorphisms were present in other breeds (PIC value from 0.080–0.371, shown in Table S5). The RIP-IDO2-14 had moderate polymorphisms in the Tibetan and Meishan breeds, with PIC values equal to 0.332 and 0.375, respectively (Table S5), but all had homozygous insertion in other breeds. In this study, we selected RIP-CWH43-9, RIP-IDO2-9, RIP-OPN-1, RIP-PRLR-6, and RIP-VMP1-12 to conduct detection in 252 individuals of Large White, which had rich distinguished polymorphisms in the breed of Large White pigs and all predicted SINEA insertion in different pig breeds with three genotypes. The detailed detection results of these five RIPs in Large White and other breeds are shown in Table 3. From the present study, it was noted that RIP-CWH43-9 was only polymorphic in the Large White, SuJiang and SuShan breeds, but not others.

**Figure 3:**
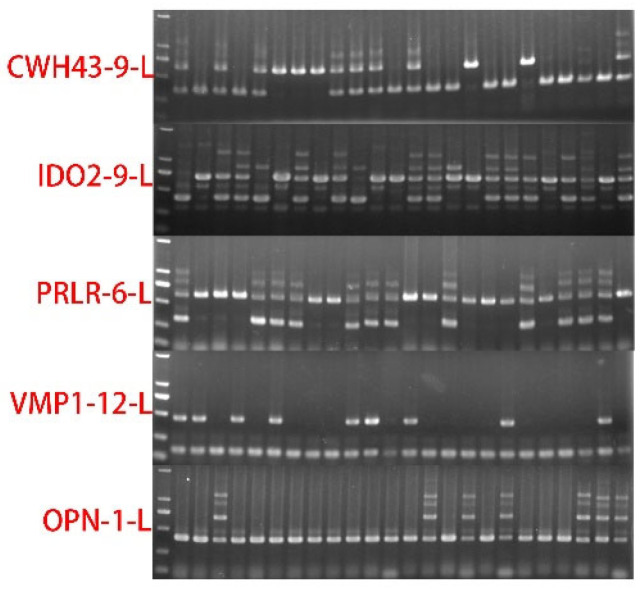
The five RIPs in the breed of Large White pigs (*n* = 24).

The effects of five RIPs on the total TNB, NBA, and LW were analyzed for Large White pigs. The electrophoresis results are presented in Figure S6, the statistical analysis results are shown in Table 4, and the detail *p*-value is shown in Table S6. We found that RIP-CWH43-9 extremely significantly (*p* < 0.01) affected the reproductive traits of TNB and NBA. Similarly, a significant effect (*p* < 0.05) on the reproductive traits of LW in Large White was also observed. The RIP-IDO2-9 significantly affected (*p* < 0.05) the reproductive traits of LW in Large White. On the other hand, there was no significant relationship between RIP-OPN-1, RIP-PRLR-6, and RIP-VMP1-12 on the reproductive traits in Large White. The PCR gel detection results for the five RIPs are shown in Figure 3.

## 4. Discussion

Structural variants (SVs) are polymorphisms involving a segment of DNA that differs between individuals, or in cancer, between a somatic and normal sample [23]. SVs are typically defined as fragments that are >50 bp in size [24]. These include large insertions (including transposons), inversions, balanced or unbalanced translocations, amplifications, deletions, and complex rearrangements, which do not fall specifically into any of these categories [25]. In this research, we compared 20 genes related to reproduction between 16 assembled pig breeds. From the results, 100 SVs (detailed in Table S1) were found and defined as retrotransposons, the majority of SINEA. Furthermore, two rounds of PCR determination were conducted to explore the authenticity of the 100 SVs. It was verified that 20 SVs had polymorphisms in several of our native pig breeds. The following research work was all based on these 20 RIPs.

Recently, many reports have established some evidence of retrotransposon insertion in the pig’s genome. Two new RIPs defined as SINEA insertions, related to fat type and lean type pig in the development of vertebrate associated gene (*VRTN*), may affect the splicing patterns of it [26]. The mouse genome contains active retrotransposon families of long and short interspersed repeats (LINEs and SINEs) that can cause germ line mutations via new insertions [27]. In livestock, the litter size is one of the most important reproductive traits and has a great impact on profitability [28]. To find the quantitative traits loci (QTL) and causal genes for these traits, several studies for linkages [29] and candidate genes [30] have been conducted using modern molecular information. Many of these major genes are involved in pig prolificacy such as the *ESR*, *FSHB*, *RBP4*, *PRLR*, *MTNR1A*, *OPN*, and *BMP* families and the *GDF9* genes have been characterized [31]. One insertion of 275-bp SINE found in the first intron of *PDIA4* gene was identified as being associated with litter size in Xiang pigs [32].

In our study, we selected 20 genes related to reproductive performance in pigs or other species with reference to the published data. A genome-wide association studies (GWAS) study showed that the *BRCA1*, *RBBP4* and *FUT1* genes were discovered in both differentially selected region (DSR) datasets related to female reproductive traits [33]. After a comparison of markers predicting litter size in different pig breeds, Kwon et al. [34] found that the average litter sizes were highly increased after the prediction of fertility using RAB2A (NC) in Yorkshire (1.57 piglets) and TPI (NC) in Duroc (3.14 piglets), respectively. The erythropoietin receptor (*EPOR*) has been shown to play an important role in fetal survival by promoting the maturation of red blood cells in many studies of uterine capacity and litter size in swine. The G allele of the *EPOR* g.705G > T SNP was associated with a greater litter size at both the first parity (*p* < 0.05) and later parities (*p* < 0.01), and this SNP was significantly more common in the prolific Chinese breeds [35]. Some QTL work indicated that genes including *GNRHR*, *RBP4*, *FSHB*, *LEP*, *BF*, and *EPOR* could be associated with litter size in different pig populations [36]. Although the PCR determination among these genes predicted RIPs by bioinformatics analysis revealed no polymorphism in domestic pig breeds, it could be used in other molecular biology experiments to distinguish between the hereditary character of different breeds in pig breeding.

Our heatmap figure revealed that the majority of the effective RIPs in our domestic pig breeds concentrated on the *A1CF*, *CWH43*, *IDO2*, *PRLR*, *OPN*, *VMP1*, and *Rab11FIP4* genes (Figure 1), with the exact confirmed RIP number (Table 1). Among the investigated RIPs, A1CF-4, CWH43-9, IDO2-13, PRLR-2, and Rab2A-2 were identified to have three types of polymorphism. Both Meishan and Erhualian were representative breeds of the Taihu Lake (TL) region of Eastern China. These breeds have been documented with exceptional prolificacy [37] and were also part of our clustered heatmap result. There was a vast difference between the highly prolific Chinese Taihu breeds and European breeds [38] in relation to the litter size. Similarly, Chinese Meishan gilts reached puberty much earlier and remained in estrus for a longer period of time than Yorkshire gilts, and Meishan sows had more corpora lutea than Yorkshire sows [39]. Meishan sows have a larger uterine capacity than Large White sows and this allows them to maintain their higher number of attached embryos throughout the gestation period [40]. The SuJiang and Sushan breeds are Chinese indigenous pig breeds hybridized with Taihu breeds and commercial varieties [23], while the breeds of Bama and Wuzhishan are well-known minipigs in China with less TNB and NBA [41,42,43].

In the current study, further variability of litter size in a Large White population was examined. Litter size is a trait of high economic relevance for pig breeding and has been under intense selection for the past decades. According to the literature, litter size has increased from an average of 11.7 live piglets in 2000 to 17.5 live piglets in 2019 [44]. This increase has also led to a surge in its variability, which is due to the positive genetic correlation between the mean litter size and its variability [45]. We collected 252 samples with clear reproductive traits including TNB, NBA, and LW. Among these 12 RIPs in Table 2, CWH43-9, IDO2-9, PRLR-6, VMP1-12, and OPN-1 had rich polymorphism in the breed of Large White pigs.

Earlier studies by Terman [46] indicated that PRLR significantly affects the litter size of pigs; however, its association signals of variants to these genes with litter size are inconsistent among different lines or groups. Some studies have also reported that non-synonymous SNPs of the *OPN* gene had an association with litter size traits in pigs [47,48,49]. With respect to the reproductive traits in the Large White pig breed, data from the present study showed no significant difference while determining the statistical analysis based on the RIP-VMP1-12, RIP-PRLR-6, and RIP-OPN-1 polymorphic types.

The *CWH43* and *VMP1* genes play crucial roles during embryonic development [50,51,52]. A GWAS in Erhualian reported that some SNPs in the gene of *CWH43* showed a significant genetic differentiation between the high-EBV and low-EBV sows [53]. A report on the porcine reproductive and respiratory syndrome (PRRS) indicated the existence of *CWH43* gene variants associated with the reproductive performance of sows infected with the PRRS virus [54]. In this study, we found that CWH43-9 with SINE insertion may be profitable to the reproductive trait of TNB, NBA, and LW, which had a higher mean ± SD than SINE^+/−^ and SINE^−/−^. The RIP-CWH43-9 with SINE^+/+^, compared to SINE^+/−^ and SINE^−/−^, extremely affected (*p* < 0.01) the reproductive traits related to TNB, NBA, and a significant difference (*p* < 0.05) in the reproductive traits was also related to LW (Table 4). In addition, RIP-CHW43-9 had a polymorphism in only the Large White, SuJiang and SuShan breeds, indicating that the population genetic structures of these three breeds may be close to each other. These results also agree with our pool-PCR detection results (Figure 1) that the breeds of Large White, SuJiang and Sushan clustered together in the PCA (Figure 2).

DNA methylation has been associated with the regulation of indoleamine 2,3-dioxygenase 2 (*IDO2*) expression and to also alter the patterns of *IDO2* expression; however, DNA methylation can also lead to a detrimental outcome during pregnancy [55]. Published studies have shown that *IDO2* also plays an important role in sustaining pregnancy in vertebrate animals [56]. Du et al. [57] reported that 140 bp deletion in the *IDO2* gene was associated with reproduction traits in Meishan pigs [57]. In the present study, the results showed a deletion in the *IDO2* gene in Tibetan and Meishan pig breeds as predicted. We defined this site as RIP-IDO2-14, and this 140 bp deletion with 83 bp was annotated as ERVI (Table S2), which has not been reported yet. The gel electrophoresis results of RIP-IDO2-14 in nine pig breeds are shown in Figure S3, but no polymorphism in Large White pigs was observed. Although RIP-IDO2-9 was discovered before RIP-IDO2-14 and was labeled as SINEA, the SINE insertion in IDO2-9 may be disadvantageous to the reproductive trait of LW, which had a lower mean ± SD than SINE^−/−^. However, a significant difference (*p* < 0.05) in the reproductive traits related to LW was observed in SINE^+/+^ compared to SINE (Table 4).

## 5. Conclusions

In summary, 20 alternative genes related to reproductive traits were designed and assembled from the genomes of different pig breeds. After conducting alignment using bioinformatics comparison software, and annotated by RepeatMasker, we acquired almost 100 predicted RIPs, which were used to carry out the first-round detection among 12 pig breeds by pool-PCR. Additionally, 12 undisputed polymorphic RIPs were also detected in Chinese indigenous and commercial pigs. After two rounds of screening, we obtained one RIP (A1CF-4) insert in the Bama, Large White, and Meishan breeds that only had a homozygous genotype, but low to moderately polymorphisms were present in other breeds, and five RIPs (RIP-CWH43-9, RIP-IDO2-9, RIP-OPN-1, RIP-PRLR-6, and RIP-VMP1-12) had a rich polymorphic in the Large White pig breed, which was used to conduct a statistical analysis with reproductive characteristics of Large White. Results from the heatmap, PCA, and statistical analysis consistently showed that the Large White, SuJiang and SuShan pig population breeds had a closer genetic structure, which conformed to the reality of the SuJiang and SuShan breeding features in China. Another discovery was that RIP-CWH43-9 had a SINE insertion that may be profitable to the reproductive traits of TNB, and NBA, which was significantly affected, and had a significant effect on the reproductive traits of LW, which was observed in Large White pigs. However, the SINE insertion in IDO2-9 may be a disadvantage to the reproductive trait of LW, which was significantly affected in Large White pigs.

## Figures and Tables

**Table 1:** The predicted large structural variations (SVs) by alignment in the reproductive related genes and their flanking regions.

Gene Name	Predicted RIPs	Confirmed RIPs
*BF_properdin*	1	0
*BRCA1*	10	0
*EPOR*	1	0
*FSHB*	2	0
*FUT1*	1	0
*GNRHR*	2	0
*IDO2*	16	5
*LEP*	1	0
*OPN (SSP1)*	2	2
*PRLR*	6	3
*Rab2A*	3	2
*RBP4*	1	0
*CASP6*	5	0
*CWH43*	9	2
*P2RX3*	1	0
*ZNF518A*	2	0
*UCHL1*	0	0
*VMP1*	18	3
*RAB11FIP4*	15	2
*A1CF*	4	1
Total Numbers	100	20

**Table 2:** The predicted RIP-site information detected in the different small group pig breeds.

Rip-Sites	Insertion Breeds	Deletion Breeds	Chr	Begin	End	Gene Structure	TE-Type	Length (bp)
A1CF-4	Duroc, MS	The rest of the species	5	98,976,497	98,976,792	Intron-10	ERVIII	295
CWH43-9	MSBJ, Wuzhishan, Rongchang, Jinhua, Berkshire, Bama, Pietrain, Large White	The rest of the species	8	38,940,696	38,940,699	Intron-15	SINEA	281
CWH43-11	The rest of the species	Berkshire, Jinhua, MSBJ, MS, Bama, Wuzhishan, Large White	8	38,945,310	38,945,752	3′flank	SINEA	442
IDO2-9	D-Ninghe, Tibetan, Bamei, Landrace, Hampshire, Berkshire	The rest of the species	17	9,326,173	9,326,195	Intron-7	SINEA	304
IDO2-14	The rest of the species	MSBJ	17	9,341,573	9,341,711	Intron-9	ERV I	138
VMP1-12	Duroc, Rongchang, D-Ninghe, Bamei, Berkshire	The rest of the species	12	35,992,635	35,992,950	Intron-7	SINEA	315
OPN-1	Pietrain	The rest of the species	8	131,078,869	131,078,870	Intron-5	SINEA	312
OPN-2	MSBJ	The rest of the species	8	131,076,528	131,076,529	3′flank	SINEA	311
PRLR-2	MS, Cross-bred, Berkshire, Jinhua	The rest of the species	16	20,643,376	20,643,377	Intron-6	SINEA	284
PRLR-3	Hampshire, Landrace	The rest of the species	16	20,642,748	20,642,943	Intron-6	SINEA	322
PRLR-6	Bama, Hampshire, Landrace, Rongchang, Wuzhishan, Pietrain	The rest of the species	16	20,630,123	20,630,134	Intron-9	SINEA	290
RAB11FIP4-1	Bama	The rest of the species	12	43,475,928	43,475,927	5′flank	SINEA	299

**Table 3:** The analysis of the genetic diversity of SINE-RIPs in different breeds.

RIPs	Breed	Account	Genotype Frequency (%)			Allele Frequency (%)		Hard-Weinberg Equilibrium		PIC
			+/+	+/−	−/−	+	−	X^2^	*p*	
CWH43-9	Bama	24	100	0	0	100	0	*N*	*N*	0
	Tibetan	24	100	0	0	100	0	*N*	*N*	0
	Duroc	24	0	0	100	0	100	*N*	*N*	*N*
	Large White	260	16.92	52.31	30.77	43.08	56.92	1.15	0.283	0.37
	SuJiang	24	54.17	41.67	4.16	75	25	0.624	0.43	0.282
	SuShan	24	0	41.67	58.33	20.83	79.17	18.857	<0.01	0.117
	Erhualian	24	100	0	0	100	0	*N*	*N*	0
	Meishan	24	100	0	0	100	0	*N*	*N*	0
	FengJing	24	100	0	0	100	0	*N*	*N*	0
IDO2-9	Bama	24	0	0	100	0	100	*N*	*N*	*N*
	Tibetan	24	0	54.17	45.83	27.08	72.92	28.091	<0.01	0.141
	Duroc	24	12.5	54.17	33.33	39.58	60.42	22.403	<0.01	0.227
	Large White	260	28.85	56.92	14.23	57.31	42.69	6.93	0.008	0.370
	SuJiang	24	20.83	29.17	50	35.42	64.58	28.735	<0.01	0.241
	SuShan	24	58.33	41.67	0	79.17	20.83	*N*	*N*	0
	Erhualian	24	0	8.33	91.67	4.17	95.83	0.045	0.831	0.08
	Meishan	24	0	25	75	12.5	87.5	6.083	0.014	0.095
	FengJing	24	20.83	54.17	25	47.92	52.08	18.622	<0.01	0.29
OPN-1	Bama	24	0	0	100	0	100	*N*	*N*	*N*
	Tibetan	24	0	0	100	0	100	*N*	*N*	*N*
	Duroc	24	100	0	0	100	0	*N*	*N*	0
	Large White	260	5.77	43.85	50.38	27.7	72.31	2.373	0.123	0.320
	SuJiang	24	0	0	100	0	100	*N*	*N*	*N*
	SuShan	24	0	12.5	87.5	6.25	93.75	0.488	0.485	0.083
	Erhualian	24	100	0	0	100	0	*N*	*N*	0
	Meishan	24	0	0	100	0	100	*N*	*N*	*N*
	FengJing	24	0	100	0	50	50	*N*	*N*	0
PRLR-6	Bama	24	100	0	0	100	0	*N*	*N*	0
	Tibetan	24	0	0	100	0	100	*N*	*N*	*N*
	Duroc	24	66.67	33.33	0	83.33	16.67	*N*	*N*	0
	Large White	258	59.69	37.60	2.71	78.49	21.51	3.317	0.069	0.281
	SuJiang	24	37.5	50	12.5	62.5	37.5	5.695	0.017	0.373
	SuShan	24	20.83	54.17	25	47.92	52.08	18.622	<0.01	0.29
	Erhualian	24	0	0	100	0	100	*N*	*N*	*N*
	Meishan	24	0	0	100	0	100	*N*	*N*	*N*
	FengJing	24	25	50	25	50	50	18.667	<0.01	0.305
VMP1-12	Bama	24	0	0	100	0	100	*N*	*N*	*N*
	Tibetan	24	54.17	45.83	0	77.08	22.92	*N*	*N*	0
	Duroc	24	66.67	33.33	0	83.33	16.67	*N*	*N*	0
	Large White	247	12.55	27.13	60.32	29.12	73.89	38.695	<0.01	0.277
	SuJiang	24	54.17	45.83	0	77.08	22.92	*N*	*N*	0
	SuShan	24	83.33	16.67	0	91.67	8.33	*N*	*N*	0
	Erhualian	24	58.33	41.67	0	79.17	20.83	*N*	*N*	0
	Meishan	24	0	0	100	0	100	*N*	*N*	N
	FengJing	24	25	75	0	62.5	37.5	*N*	*N*	0

Notes: +/+: SINE^+/+^ (SINE insertion homozygous genotype); +/−: SINE^+/−^ (SINE insertion heterozygous genotype); −/−: SINE^−^^/−^ (SINE no insertion homozygous genotype). +: SINE^+^ (SINE insertion allele); −: SINE^−^ (SINE no insertion allele). When *p* > 0.05, it indicates the genetic balance of the population, and the data come from the same Mendel population. PIC: Polymorphic information content. When PIC > 0.5, the locus is highly polymorphic; when 0.25 < PIC < 0.5, the locus is moderately polymorphic; when PIC < 0.25, the locus is low polymorphic.

**Table 4:** The results of the statistical analysis for the RIPs with the reproductive characteristics of Large White pigs.

RIP Name	Genotype	TNB	NBA	LW
CWH43-9	SINE^+/+^ (*n* = 43)	10.84 ± 3.14 ^A^	10.49 ± 2.95 ^A^	14.00 ± 4.12 ^A^
	SINE^+/−^ (*n* = 131)	9.61 ± 2.84 ^B^	9.15 ± 2.79 ^B^	12.42 ± 3.97 ^B^
	SINE^−/−^ (*n* = 78)	9.88 ± 2.94 ^B^	9.47 ± 2.83 ^ab^	12.67 ± 3.71 ^B^
IDO2-9	SINE^+/+^ (*n* = 72)	9.51 ± 3.21 ^a^	9.08 ± 3.03 ^a^	11.99 ± 4.11 ^a^
	SINE^+/−^ (*n* = 144)	9.9 ± 2.86 ^a^	9.51 ± 2.79 ^a^	12.93 ± 3.89 ^ab^
	SINE^−/−^ (*n* = 36)	10.58 ± 2.64 ^a^	10.11 ± 2.73 ^a^	12.77 ± 3.78 ^b^
PRLR-6	SINE^+/+^ (*n* = 168)	9.98 ± 2.91 ^a^	9.55 ± 2.84 ^a^	12.88 ± 4.07 ^a^
	SINE^+/−^ (*n* = 77)	9.79 ± 3.05 ^a^	9.36 ± 2.91 ^a^	12.55 ± 3.75 ^a^
	SINE^−/−^ (*n* = 7)	9.92 ± 2.81 ^a^	8.86 ± 3.24 ^a^	12.41 ± 4.03 ^a^
VMP1-12	SINE^+/+^ (*n* = 31)	9.34 ± 2.64 ^a^	9.61 ± 2.59 ^a^	12.68 ± 3.57 ^a^
	SINE^+/−^ (*n* = 65)	10.34 ± 2.85 ^a^	9.92 ± 2.68 ^a^	13.19 ± 3.65 ^a^
	SINE^−/−^ (*n* = 145)	9.68 ± 3.10 ^a^	9.24 ± 3.04 ^a^	12.57 ± 4.24 ^a^
OPN-1	SINE^+/+^ (*n* = 15)	9.80 ± 2.81 ^a^	9.67 ± 2.74 ^a^	12.54 ± 3.63 ^a^
	SINE^+/−^ (*n* = 108)	9.93 ± 2.94 ^a^	9.59 ± 2.84 ^a^	12.95 ± 4.02 ^a^
	SINE^−/−^ (*n* = 129)	9.90 ± 2.98 ^a^	9.43 ± 2.91 ^a^	12.64 ± 3.97 ^a^

Note: This statistical analysis was processed by the nonparametric test pairwise comparison of independent samples. The same letter in the same column of the means ± SD indicates that the difference between groups was not significant. Different superscript lowercase letters indicate a significant difference between groups (*p* < 0.05). Different superscript capital letters indicate an extremely significant difference between groups (*p* < 0.01).

## Data Availability

All data needed to evaluate the conclusions in this paper are present either in the main text or the Supplementary Materials.

## Supplementary Materials

The following supporting information can be downloaded at: https://www.mdpi.com/article/10.3390/genes13081359/s1. Figure S1: PCR combine; Figures S2–S5: The PCR result of 24 individuals’ detection per breeds based on RIPs; Figure S6: The results of RIPs in the breed of LargeWhite pigs. Table S1: Assemble information for 20 reproduction genes from the 16 pig genomes; Table S2: Reproduction genes SVs prediction; Table S3: PCR results convert to numbers; Table S4: The PCR result of 24 individuals’ detection per breeds based on RIPs convert to numbers; Table S5: Polymorphism detection results of the 12 SINE insertion in different breeds; Table S6: The *p*-value result of statistical analysis for the RIPs with the reproductive characteristics of Large White pigs.
