# Supplementary figures and images for: Retrotransposon Insertion Polymorphisms (RIPs) in Pig Reproductive Candidate Genes
Source: Genes (Basel). 2022 Jul 28;13(8):1359. doi: 10.3390/genes13081359 (PMC9407582; doi:10.3390/genes13081359)

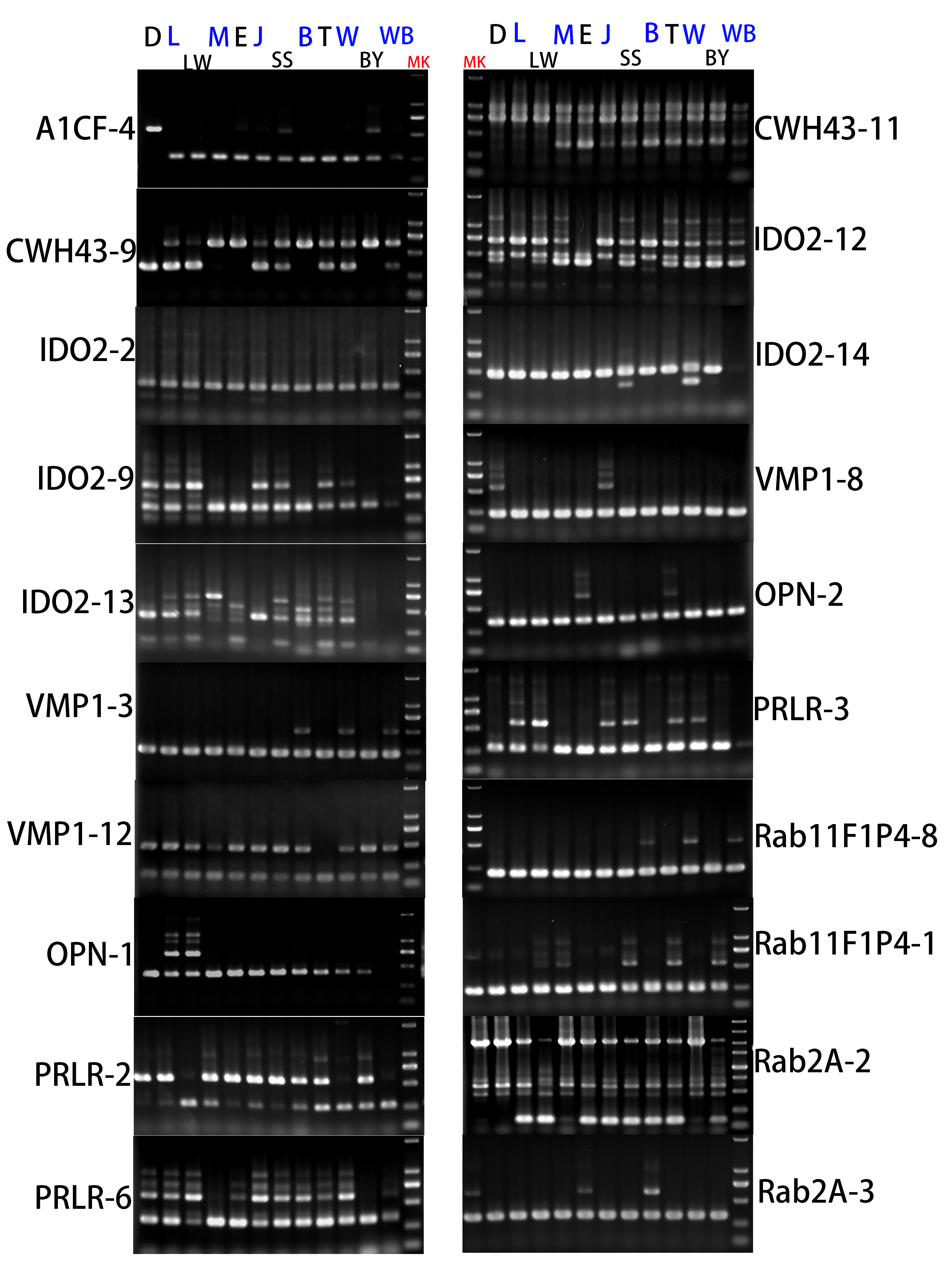

Supplement: Supplementary file 1 [file genes-13-01359-s001.zip › supplemental files/Figure S1. PCR combine.tif]

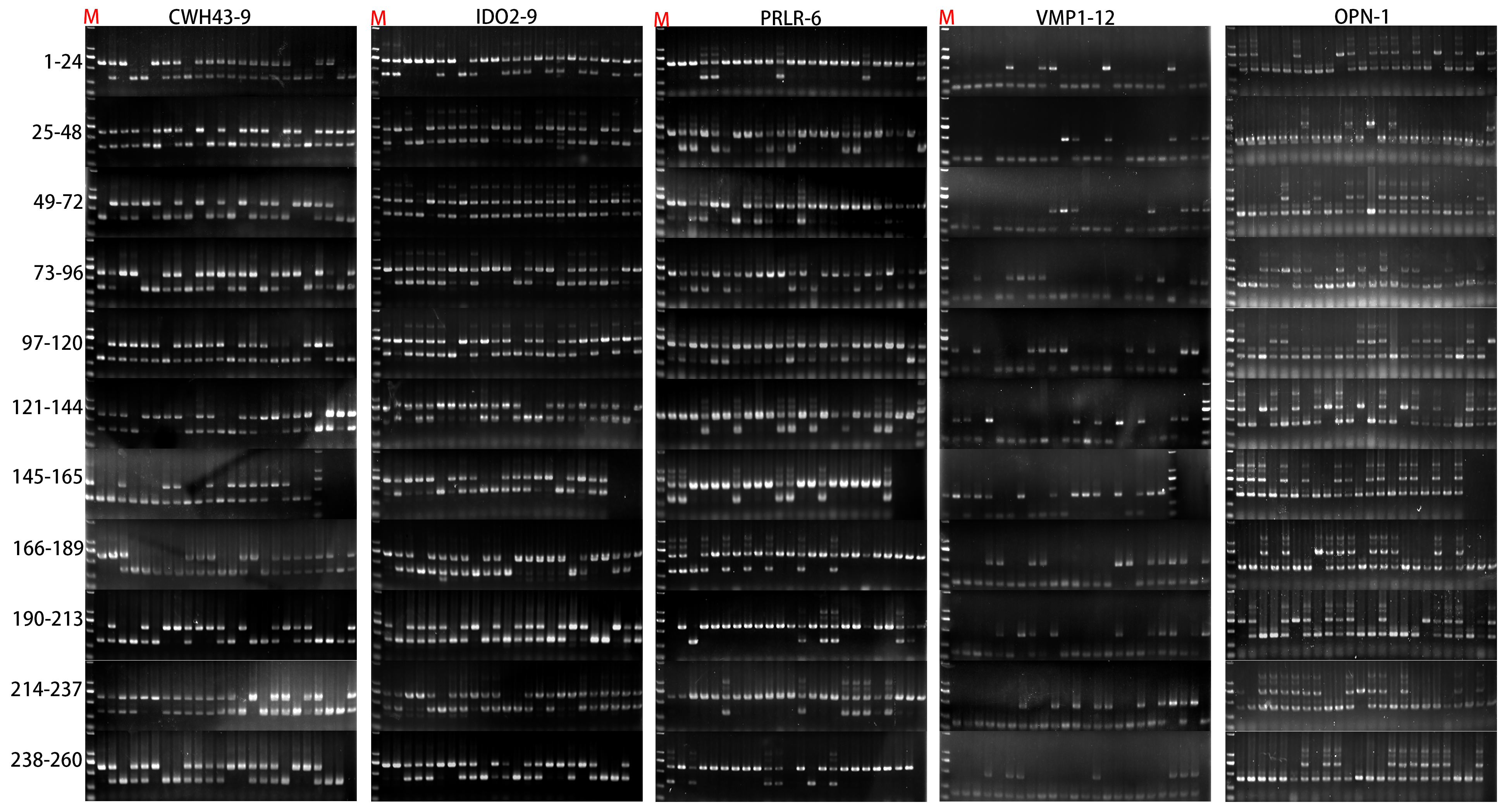

Supplement: Supplementary file 1 [file genes-13-01359-s001.zip › supplemental files/Figure S6. The PCR result of 24 individuls detection per breeds based on RIPs .tif]
